# Body reserves mediate trade-offs between life-history traits: new insights from small pelagic fish reproduction

**DOI:** 10.1098/rsos.160202

**Published:** 2016-10-05

**Authors:** Pablo Brosset, Josep Lloret, Marta Muñoz, Christian Fauvel, Elisabeth Van Beveren, Virginie Marques, Jean-Marc Fromentin, Frédéric Ménard, Claire Saraux

**Affiliations:** 1 University of Montpellier, UMR MARBEC (IRD, Ifremer, UM, CNRS), 34203 Sète, France; 2 IFREMER, UMR MARBEC (IRD, Ifremer, UM, CNRS), 34203 Sète, France; 3 Faculty of Sciences, University of Girona, Girona, Spain; 4 IFREMER, UMR MARBEC (IRD, Ifremer, UM, CNRS), 34250 Palavas Les Flots, France; 5 IRD, Mediterranean Institute of Oceanography (MIO), Aix-Marseille Université/CNRS/IRD/Université de Toulon, UM 110, 13288 Marseille, France

**Keywords:** maternal effect, northwest Mediterranean, anchovy, sardine

## Abstract

Limited resources in the environment prevent individuals from simultaneously maximizing all life-history traits, resulting in trade-offs. In particular, the cost of reproduction is well known to negatively affect energy investment in growth and maintenance. Here, we investigated these trade-offs during contrasting periods of high versus low fish size and body condition (before/after 2008) in the Gulf of Lions. Female reproductive allocation and performance in anchovy (*Engraulis encrasicolus*) and sardine (*Sardina pilchardus*) were examined based on morphometric historical data from the 1970s and from 2003 to 2015. Additionally, potential maternal effects on egg quantity and quality were examined in 2014/2015. After 2008, the gonadosomatic index increased for sardine and remained steady for anchovy, while a strong decline in mean length at first maturity indicated earlier maturation for both species. Regarding maternal effects, for both species egg quantity was positively linked to fish size but not to fish lipid reserves, while the egg quality was positively related to lipid reserves. Atresia prevalence and intensity were rather low regardless of fish condition and size. Finally, estimations of total annual numbers of eggs spawned indicated a sharp decrease for sardine since 2008 but a slight increase for anchovy during the last 5 years. This study revealed a biased allocation towards reproduction in small pelagic fish when confronted with a really low body condition. This highlights that fish can maintain high reproductive investment potentially at the cost of other traits which might explain the present disappearance of old and large individuals in the Gulf of Lions.

## 1. Introduction

Reproduction and maintenance require important and often conflicting energy investments in all animal species [1,2]. Further, additional constraints such as limited resources or physiological constraints might also limit the energy available to be allocated, preventing simultaneous maximization of all life-history traits and bringing out trade-offs [3,4]. In particular, the main trade-off involves the cost of reproduction [3], represented by negative correlations between the current reproductive effort and both maintenance and future reproduction [5]. In a situation of food shortage and low individual energy reserves, this might lead to extreme choices, such as either temporarily stopping reproduction (skipping of breeding event) or maintaining reproduction at the cost of survival [6].

Such energy allocation trade-offs are known to be affected by the environment, whether social [7] or physical [8], but also by parent's phenotype [9]. In particular, parent size or condition may strongly affect individuals' resolution of the trade-off (e.g. birds [10], mammals [11] and fish [12]), resulting in an effect of parental phenotype on reproductive output (e.g. increase of female relative fecundity with increasing body size in fish [13], turtles [14] or *Daphnia* [15]). This is generally known as the maternal effect, i.e. when the phenotype of an organism is influenced not only by its own genes and the environment, but also by the characteristics of its mother, which might increase or decrease the chance of survival for the offspring. In addition, studies on diverse species have demonstrated that young females may not produce eggs of the same quality [15,16] or as many eggs as older females per unit of maternal biomass (i.e. relative fecundity, [17]). Besides a decrease in size and age, a drop in body condition might also influence reproduction. Condition is commonly considered as the quantity of nutrient reserves and a proxy of fitness [18], determining the survival and reproductive capacity of individuals and populations. Indeed, energy is usually the main limiting factor preventing individuals from maximizing life-history traits [19–21]. Some studies have shown that a better body condition of the females may allow the allocation of energy surplus to reproduction (increasing reproductive outputs in quantity and quality), thereby influencing population growth [22–24]. By contrast, species have been observed to reduce their reproductive potential (e.g. birds [25] or fish [26]) or skip the spawning period (e.g. turtle [27] or fish [28]) in situation of low reserves. Those contrasted findings usually fit the life-history predictions that species with slower life-history paces should allocate more into survival and future reproduction, while short-lived species should dedicate most of their energy towards reproduction [3].

Small pelagic fish are relatively short-lived species, known to commonly face strong variations in abundance and biomass [29]. In particular, anchovy and sardine are important forage fish, which form a key component of the pelagic ecosystems [30,31], and constitute the prey of numerous predators (e.g. tuna, marine mammals and seabirds). Small pelagic fish also support the most important fisheries in the world and employ a significant number of people [32]. The ratio of biomass to abundance (i.e. the mean population weight) of the anchovy (*Engraulis encrasicolus*) and sardine (*Sardina pilchardus*) populations in the Gulf of Lions (northwestern Mediterranean Sea) has dramatically decreased since 2008, while a pronounced increase in both abundance and biomass of another small pelagic fish species, namely sprat (*Sprattus sprattus*) has occurred simultaneously [33]. The decline went along with a marked decrease in size, age and condition as well as changing diet for both species [33]. This raised several questions regarding (i) the resolution of the reproduction versus maintenance trade-off in a situation of low energetic reserves and (ii) the effects on anchovy and sardine reproductive capacities and ultimately population dynamics.

Anchovy and sardine share the Gulf of Lions as an important spawning area, but their spawning characteristics and habitats are clearly differentiated [34]. On the one hand, anchovy reproduces during late spring and summer when water temperatures reach values between 17°C and 23°C and is an income breeder [34,35]. On the other hand, sardine is mainly known as a capital breeder reproducing in cold water (temperatures ranging between 12°C and 14°C) from December to March [36,37]. Despite their opposite reproductive strategies, the two species are batch spawners (i.e. they release eggs in batches over a protracted spawning season, [38,39]). While the trade-off between somatic and reproductive functions has been long considered [3,5,19,40], especially in the context of the slow–fast life-history gradient, these two species thus offer a unique opportunity to investigate the cost of reproduction in a situation of food shortage in two species sharing very similar characteristics but for their capital versus income breeding strategies.

The main purpose of this study was, on the one hand, to investigate the trade-off between the different life-history traits and, on the other hand, to assess how small pelagic fish reproductive outputs fluctuated. To do so, we used length at first maturity (*L*_50_), the gonadosomatic index (GSI) and reproductive period durations as indices of reproductive investment. We investigated their temporal changes between 2003 and 2015, a period in which drastic changes in body condition were observed, and compared them to values in the 1970s. In a second step, biometry and gonad analyses were performed over one spawning season, to investigate the effect of adult size and condition on the reproductive output (quantity and quality of eggs) of both species, i.e. the maternal effects. This helped reconstructing an index of the number of eggs spawned by the two populations from 2003 to 2016 and understanding whether the current situation of small and low-condition fish hampered the reproductive potential of the population. Sampling effort and analyses were limited to females as their reproductive capacity is the main driver of the population dynamics, caused by the high cost of egg production relative to the energy needed for producing sperm [41,42]. All these parameters are frequently considered for fisheries research and management, but are rarely reported simultaneously over a long-term period for multiple species. Linking fish condition to reproductive capacity could greatly aid the understanding of the population dynamics of sardine and anchovy. In turn, such understanding could benefit the management of their fisheries.

## 2. Material and methods

### 2.1. Fish sampling

A total of 8887 female anchovies was sampled from 2003 to 2015. Female sardines (*N* = 10 541) were caught between 1971 and 1978 (*N* = 2192) and between 2004 and 2016 (*N* = 8259). Samples were randomly collected in trawls of scientific surveys (PELMED & MEDITS, from 2003 onwards) by successively dividing the total anchovy catch until the quantity necessary for analyses was reached or obtained from commercial trawlers operating in the Gulf of Lions during all years. All morphometric analyses were conducted following the same methodology so that data from different years, periods, etc., would be comparable. Briefly, total body length (*L*_T_, to the nearest millimetre), body mass (*M*, to the nearest 0.1 g), eviscerated body mass (*M*_E_, to the nearest 0.1 g), sex and gonad mass (*M*_G_, to the nearest 0.1 g) were recorded for each individual. Maturity stages were determined by visual examination of the gonads, using a six-stage key in which stage 1 indicates immature individuals, stages 2–4 illustrate three steps of increasing development of gonads, stage 5 shows the spawning capable individuals and stage 6 features the post-spawning period [43]. Fish at stage 2 and above were considered to be adults, forming the putative spawning population. Fish in stages 3–5 were assumed to be showing reproductive activity. An additional analysis was performed on 108 sardines sampled during the 2014–2015 winter and 126 anchovies collected during the 2015 summer. Individuals were either dissected onboard (PELMED) or placed in plastic bags filled with ice to be transported to the laboratory (trawlers), where they were immediately dissected. One gonad was fixed in 4% buffered formaldehyde for histological processing and oocyte quantity and quality estimation, as recommended by Rakka & Ganias [44]. A piece of muscle was also removed and frozen at −80°C for further lipid content determination.

### 2.2. Historical changes in reproductive patterns

First, we investigated the duration and timing of reproduction along the studied years, using the monthly percentage of mature individuals (stage 5) that were determined as spawning capable from the ICES visual key [43]. A lack of data in some months for some of the years prevented accurate yearly representations. As fish population changes have been progressive [33,45], data were thus pooled into two equal time periods (2008–2011 and 2012–2015) to increase sample sizes and representativeness (*n* ≥ 50 in any given month). These breeding cycles were then compared to previously published data from 1965 [46]. Additionally, data on the mean breeding stage of sardines collected in 1959 [47] were also used for comparative purposes.

In order to confirm these results, a second approach to assess breeding phenology is presented by using GSI (see below) seasonality in the 1970s and between 2002 and 2015 for sardine and between 2003 and 2015 for anchovy.

Two measures were used to describe small pelagic fish reproductive investment. The GSI was calculated using the following formula:

$$\text{GSI}=\left(\frac{M_{G}}{M_{E}}\right)\times 100,$$

where *M*_G_ is the gonad weight and *M*_E_ the fish eviscerated weight. The use of this index was validated by confirming the isometric relationship between gonad weight and fish eviscerated weight, preventing us from misleading interpretation.

Length at first maturity (*L*_50_), i.e. body length at which 50% of the individuals were mature, was estimated per spawning season between 2003 and 2015 for anchovy and from 1971 to 1976 and 2003 to 2016 for sardine. To do so, annual maturity ogives were created per species, plotting the proportion of mature individuals relative to fish length during the spawning season. Generalized Linear Models (GLM) with a binomial error distribution and a logit link were used to approximate the ogive, with the proportion of mature fish (*m*) as the dependent variable and the length classes (*L*_C_, 0.5 cm) as the independent variable. The models had the general form:

$$\text{Logit}(E[m])=a+bL_{C},$$

where *a* and *b* are the intercept and slope of the ogive, respectively. *L*_50_ for each year was derived from the estimated parameters:

$$L_{50}=-a/b,$$

### 2.3. Fish muscle lipid content

Fish condition was estimated by muscle lipid content for anchovy and sardine collected during their spawning season, respectively, in the 2015 summer and the 2014–2015 winter. Liver was not investigated here as its weight was too small in comparison with the weight of other organs (less than 1%), and these two species are renowned for storing lipids in the muscle [12]. Roughly 0.1 g of muscle was sampled in order to extract lipids using a solvent mixture (chloroform–methanol 2 : 1, v/v), as described by Folch *et al*. [48] and analysed by flame ionization detection on an Iatroscan [49]. For the purpose of this study, only total lipid content obtained by summation of individual lipid classes is presented.

### 2.4. Reproductive capacity

#### 2.4.1. Histological determination and follicular atresia

These analyses were made only on samples from the 2015 summer (anchovy) and the 2014–2015 winter (sardine). After fixation, one gonad was cut transversely along its midsection and embedded in paraffin before being sliced into 5–10 µm sections and stained with both haematoxylin-eosin and Mallory's trichrome stains. The latter staining method highlights the zona radiata and its continuity and facilitates the detection of degenerating oocytes which will not be spawned, i.e. atretic oocytes [50].

**Figure 1.**
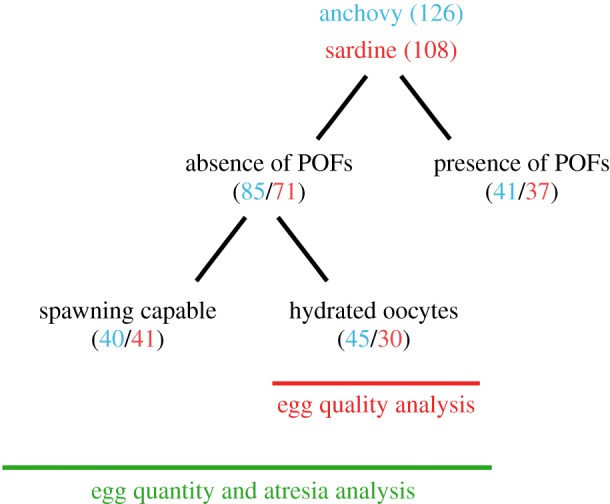
Sample size for fecundity analyses for anchovy (blue) and sardine (red).

Histological analyses allowed us to select individuals used in fecundity and egg quality analyses. First, we used the terminology employed by Brown-Peterson *et al*. [51] to histologically describe the developmental stage of the oocytes; immature (fish that have not reached sexual maturity), regenerating (mature but reproductively inactive individuals), developing (fish with gametes that are beginning to develop), spawning capable (fish with advanced gametes that are ready for spawning), actively spawning (oocytes in migratory nucleus or hydration stage) or regressing (massive atresia which indicates the end of the reproductive cycle). Only fish in the actively spawning stage were retained for later analyses on egg quality, whereas both spawning capable and actively spawning stages were kept for estimations of batch fecundity (BF). To avoid underestimations of BF, histological analyses were used to check for the presence of postovulatory follicles, which reveal if spawning had already started. Egg quality analyses were performed with hydrated oocytes. For the experimental part of this study, 108 female sardines were analysed, including 41 individuals classified as spawning capable and 30 individuals with hydrated eggs. Of the 126 female anchovy, 45 individuals held gonads with hydrated oocytes and 40 were retained as spawning capable individuals (figure 1).

In a second step, histological analyses were also performed to study atresia. Atresia were quantified with two different measures: the prevalence of atresia (*P*_a_), calculated as the proportion of females with α-atretic oocytes, and the relative intensity of atresia (*I*_a_), determined for females exhibiting atretic oocytes as the number of α-atretic oocytes divided by the total number of vitellogenic oocytes. Three different gonad areas were analysed for both indices and the mean of the three areas was used as the relative intensity of atresia.

#### 2.4.2. Fecundity and egg quality

To assess fecundity, subsamples of the central part of the ovary were weighed before being washed to separate the oocytes from the connective tissue [52]. Anchovy and sardine are batch spawners, so their fecundity was estimated in terms of BF, defined as the number of eggs spawned per batch [53]. Three sieves with a mesh size ranging from 600 to 250 µm were used to sort the oocytes by size. Oocyte size distribution followed a bimodal distribution and eggs belonging to the next batch were counted based on their size as defined in [39,54] for anchovy. Counting of oocytes was performed with an image analysis system (Image-Pro Plus 5.1; http://www.mediacy.com). The BF was estimated with the following formula:

$$\text{BF}=M_{G}\times \left(\frac{O}{S_{W}}\right),$$

where *M*_G_ is the gonad weight after fixation, *O* is the number of oocytes counted in the subsample of the ovary and *S*_W_ is the subsample weight. In addition, the relative batch fecundity (RBF) was computed as the BF divided by the eviscerated weight of the fish (g) and was formerly validated following the same procedure as for the GSI.

Oocyte quality, another proxy of reproductive success [55], was assessed by estimating the mean oocyte dry mass for each fish, obtained by drying two replicates of 100 hydrated oocytes for 24 h at 110°C.

#### 2.4.3. Estimation of the number of eggs spawned by the populations

An estimation of the total population egg production for each year and species was calculated by combining (i) the number of fish per length class in the population, (ii) maturity ogives, (iii) length-dependent BF, and (iv) the number of spawning events (or batches) per length class during the spawning season in the same formula:

$$\text{egg}\;\text{number}=\underset{li}{\sum }\left(n_{li}\times M_{li}\times \text{B}{\text{F}}_{li}\times \frac{\text{spawning }\;\text{duration}}{\text{batch }\;\text{perio}{\text{d}}_{li}}\right).$$

With *n_*li*_* the number of fish in length class *l_*i*_*, *M_*li*_* the percentage of mature individuals in length class *l_*i*_*, BF*_*li*_* the BF for length class *l_*i*_* and spawning duration/batch period_*li*_ the number of spawning events for length class *l_*i*_*.

Here *n_*li*_* was obtained from PELMED acoustic surveys, which took place during summer (i.e. the reproductive period of anchovy). However, as sardines reproduce between December and March, size structure information needed to be corrected for this species. Therefore, the theoretical sizes of sardine in winter were estimated from their sizes of the preceding summer recorded during PELMED, using a growth correction formula. Given that small pelagic fish grow mainly during summer, seasonal growth variability was accounted for using Somers' model based on 2003–2014 otolith data [56,57]. Adult mortality was assumed to be size independent between summer and the beginning of winter, as mortality was assumed to occur mostly in the first weeks of life or during overwintering. *M_*li*_* was obtained from annual maturity ogives (see Historical changes in reproductive patterns), while BF*_*li*_* was inferred from fecundity analyses (see Fecundity and egg quality).

The number of spawning events was obtained by combining the spawning duration (obtained from annual reproduction cycles depending on the studied period; see Historical changes in reproductive patterns) and the between-batches period. The latter was size dependent and obtained for sardine from [58]: individuals smaller than 13 cm spawn every 17 days, those ranging between 13 and 16 cm spawn every 12.25 days and those greater than 16 cm spawn every 7.81 days. Regarding anchovy, the spawning frequency changes with age and was derived from dynamic energy budget (DEB) modelling [59,60]. This number of batches from DEB modelling is close to direct observations [59]. Age was transformed into size using mean length at age [33]. Owing to the size decrease during the last decade, separate values were determined before and after 2008. Therefore, age 1 anchovy spawning every 5.26 days corresponds to a size range between 11.5 and 13 cm before 2008, and between 10 and 11.5 cm after 2008. Age 2 individuals spawning every 4.35 days fall into a size range of 13–15 cm before 2008, and 11.5–14.5 cm after 2008. Age 3 anchovy spawning every 4.17 days has a size superior to 15.5 cm before 2008 and 14.5 cm after 2008 [33]. Egg quality was not included in this simulation because of the difficulties to link morphometric to biochemical condition measurements during the reproductive period [49].

### 2.5. Data analyses

We conducted linear regression analyses between total muscle lipid content and both egg quantity and quality. The coefficient of determination (*R*^2^) was used to estimate the proportion of variability explained and the strength of the relationship between the different variables tested. Similarly, linear regressions were also carried out per species between the intensity of atresia and the muscle lipid content and fish length. GLMs with a binomial error distribution were conducted to investigate the relationships between fish length or condition and atresia prevalence. For all relationships and metrics (e.g. GSI, RBF), residuals were tested for normality and homogeneity of variance, and transformed if necessary.

GSI annual cycles (period 1973–1978 plus 2002–2015 for sardine and 2004–2015 for anchovy) were used to generate GSI anomalies calculated as the difference between the observed GSI of each fish for a given month, and the mean GSI of that month over the entire dataset. Then, in order to investigate interannual variation in GSI, the analyses were restricted to the spawning months where at least 25% of the population was spawning (as defined in the results on breeding phenology). For both species, GSI interannual variability and differences between time periods were tested using one-way analyses of variance (ANOVA and pairwise post-hoc tests).

For the calculation of the number of eggs spawned annually per species, 1000 simulations were performed in order to incorporate the uncertainties derived from fecundity and maturity at length as well as spawning duration. Parameters for which an estimation of the associated uncertainty was available (i.e. *M_*li*_*, BF*_*li*_*) were randomly drawn from a uniform distribution whose range was defined by the 95% confidence interval calculated from the size–maturity and size–BF GLM results. Spawning duration was also randomly drawn according to the range inferred from results of the reproduction annual cycles. All statistical analyses were performed with R v. 3.0.2 (R Development Core Team, 2013). Values are indicated as mean ± s.e. and all statistical tests were performed at a significance level of 0.05.

## 3. Results

### 3.1. Historical perspective of reproductive patterns

**Figure 2.**
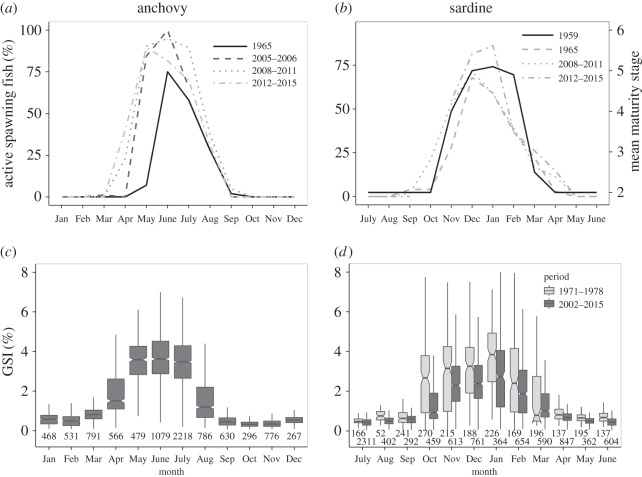
Breeding cycles in different years or periods for anchovy (left side) and sardine (right side). The top panels represents the proportion of mature females in stage 5 (i.e. individuals actively spawning or close to spawn), except for 1959 data on sardines (adapted from [47]) which shows the mean maturity stage in all mature females. Data from 1965 (sardines and anchovies) are derived from [46]. The bottom panels display the GSI index.

Based on the percentage of spawning capable individuals, anchovy spawning season lasted longer (five months) and started earlier (between April and August, figure 2*a*) during the two recent time periods (2008–2011 and 2012–2015) than in 1965 or 2005–2006 (three or four months between May–June and August; figure 2*a*). The GSI also indicated that anchovy reproduction in recent years was limited to the April–August period (figure 2*c*). By contrast, sardine spawning season remained relatively stable over the different time periods, lasting five months from November to March (figure 2*b*). Nonetheless, small variations were registered in 2008–2011, when the season started slightly earlier, as about 25% of the population was already spawning in October. Also, a small part of the population (approx. 10–15%) was still spawning in April during the two recent periods (2008–2011 and 2012–2015). The sardine spawning season also lasted from October to March in the 1970s and 2002–2015, according to annual GSI cycle (figure 2*d*).

**Figure 3.**
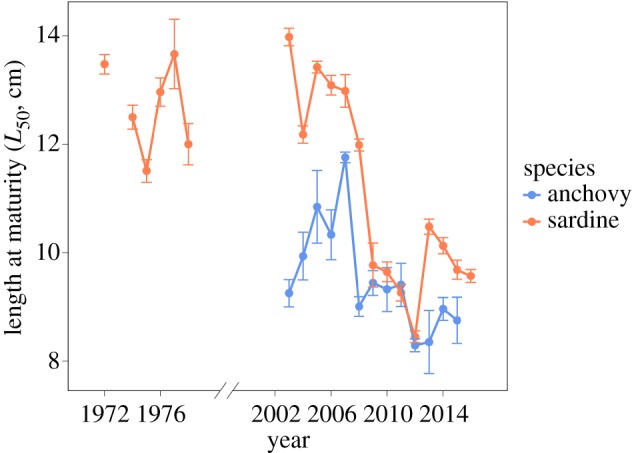
Interannual variability in length at first maturity (*L*_50_) between 2003 and 2015 for anchovy and sardine.

Length at maturity (*L*_50_) estimated for the spawning seasons 2002–2015 using yearly GLMs showed strong interannual variations (figure 3). Results for anchovy indicated that *L*_50_ first increased from 2003 to 2007, then strongly declined in 2008 and at last stabilized around 9.3 cm. For sardine, a major downward shift in *L*_50_ was observed during the late 2000s, from 12.1 cm during 2002–2008 to 9.6 cm after 2009.

**Figure 4.**
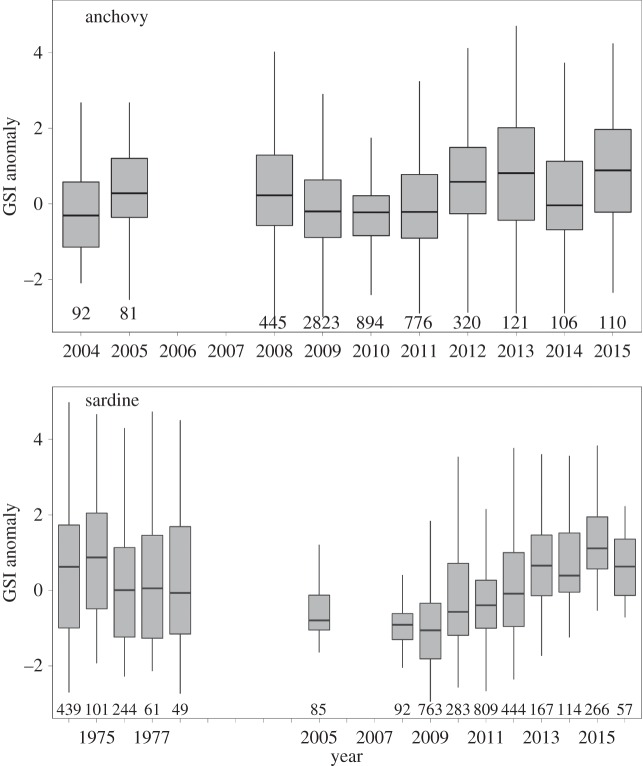
Interannual variability in GSI anomaly values for anchovy (2004–2015) and sardine (1974–2015).

Anchovy GSI anomalies exhibited significant variations between years (*p* < 0.001, figure 4), with an alternation of negative and positive anomalies, and a slight increase between the two periods (2004–2005 versus 2008–2015, *p* < 0.01). Sardine GSI anomalies also significantly fluctuated between years (*p* < 0.001, figure 4). In the 1970s, GSI anomalies fluctuated around small positive values without any temporal trend. By contrast, during the more recent period a clear significant rise (linear model, *p* < 0.001) in GSI anomalies values appeared, from strong negative values in 2008/2009 (mean = −0.81/−0.99) to the highest values in the series in 2015 (mean = 1.73).

### 3.2. Effects of size and condition on batch fecundity and atresia

**Figure 5.**
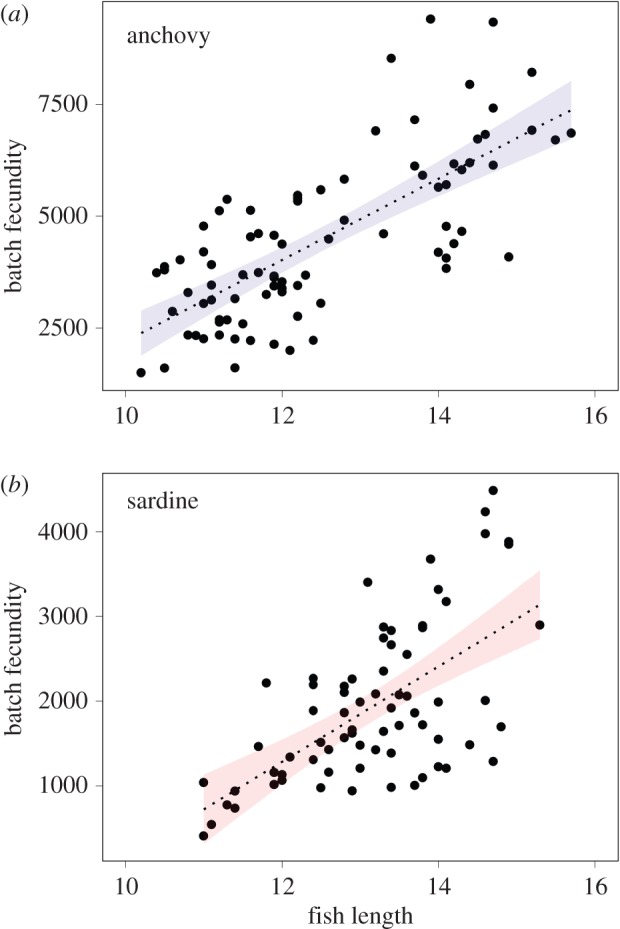
Relationship between BF and fish length for anchovy and sardine. Lines indicate significant linear regressions, while shaded zones correspond to the 95% CI.

Female anchovy egg numbers ranged from 1492 to 9406 eggs per batch, whereas sardine ovaries had between 537 and 4486 eggs per batch. Both species exhibited a significant and positive relationship between gonad fresh weight and BF (egg number: *n* = 85, *R*^2^ = 0.61, *p* < 0.001 for anchovy and *n* = 71, *R*^2^ = 0.34, *p* < 0.001 for sardine). BF was also positively related to the total length of fish (*n* = 85, *R*^2^ = 0.53, *p* < 0.001 for anchovy and *n* = 71, *R*^2^ = 0.38, *p* < 0.001 for sardine; figure 5) as was the relative BF, although not significantly for anchovy (*n* = 85, *R*^2^ = 0.05, *p* = 0.07 for anchovy and *n* = 71, *R*^2^ = 0.10, *p* = 0.03 for sardine). On the contrary, neither BF (*n* = 85, *p* = 0.07 for anchovy and *n* = 71, *p* = 0.24 for sardine) nor RBF (*n* = 85, *p* = 0.67 for anchovy and *n* = 71, *p* = 0.12 for sardine) was related to muscle lipid content. Anchovy ovaries with atretic oocytes had a low prevalence (prevalence of atresia, *P*_a_ = 17.8 and 22.5; table 1) and intensity (relative intensity of atresia, *R*_Ia_ = 13.1 and 16.9; table 1), for both spawning capable and actively spawning stages. Similar observations were made for sardine, with atresia prevalence ranging from 29.3% for spawning capable individuals to 6.7% for actively spawning individuals (table 1). Intensity of atresia was slightly lower than for anchovy, with values from 13.5% for spawning capable individuals to 3.4% for actively spawning individuals (table 1). No significant relationship between prevalence or intensity of atresia and both fish condition and length was found for either species (table 1).

**Table 1.** Prevalence of atresia (*P*_a_, %) and mean relative intensity of atresia (*R*_Ia_, %) for both stages of ovarian development studied in anchovy and sardine. *R*_Ia_ was estimated on fish presenting atresia only to avoid incorporating a large number of 0. Here *n* represents the total number of fish on which atresia was investigated; *p*-values of the relationships between fish *P*_a_ or *R*_Ia_ and fish condition and length are given in the table; n.a. indicates that a statistical analysis was not performed due to a too low sample size.

species	maturity stage	*n*	*P*_a_ (%)	condition versus *P*_a_	length versus *P*_a_	mean *R*_Ia_ (%)	condition versus *R*_Ia_	length versus *R*_Ia_
anchovy	spawning capable	40	22.5	0.26 (n.s.)	0.28 (n.s.)	13.1	0.56 (n.s.)	0.81 (n.s.)
	actively spawning	45	17.8	0.71 (n.s.)	0.54 (n.s.)	16.9	0.67 (n.s.)	0.90 (n.s.)
sardine	spawning capable	41	29.3	0.81 (n.s.)	0.60 (n.s.)	13.5	0.77 (n.s.)	0.68 (n.s.)
	actively spawning	30	6.7	0.30 (n.s.)	0.95 (n.s.)	3.4	n.a.	n.a.

### 3.3. Effects of size and condition on egg quality

**Figure 6.**
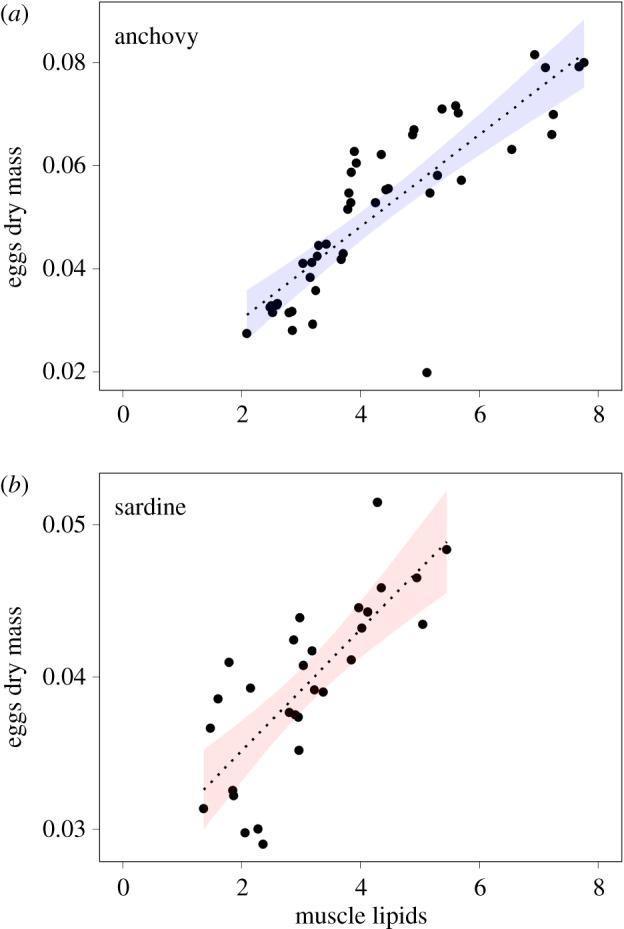
Relationship between egg quality and fish condition measured as the total lipid content in muscle for anchovy and sardine. Lines indicate significant linear regressions, while shaded zones correspond to the 95% CI.

Mean egg dry mass was established for hydrated females only, and estimated at 0.052 (±0.016) and 0.039 (±0.006) mg per egg for anchovy and sardine, respectively. For both species, this index of egg quality was positively correlated with fish muscle lipid content, but more strongly for anchovy (*n* = 45, *R*^2^ = 0.71, *p* < 0.001; figure 6) than for sardine (*n* = 30, *R*^2^ = 0.59, *p* < 0.001; figure 6). No relationship was found between egg quality and fish size for either species (*n* = 45, *p* = 0.10 and *n* = 30, *p* = 0.21 for anchovy and sardine, respectively).

### 3.4. Number of eggs spawned by small pelagic fish

**Figure 7.**
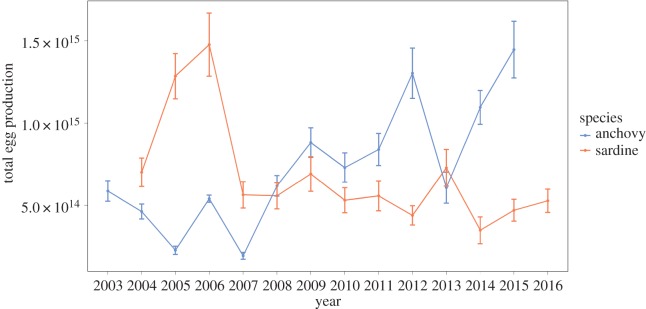
Total egg number spawned in the Gulf of Lions between 2003 and 2016 for anchovy and sardine. Error bars represent the standard error associated to each year.

To calculate the annual total number of eggs produced per population, anchovy spawning season ranged from three to five months, based on previous results. Simulations for sardine were realized for a spawning season duration set to five or six months. Anchovy egg number production showed large interannual fluctuations with a low egg number between 2004 and 2008 (between 4.63 × 10^14^ and 6.18 × 10^14^ eggs, figure 7) while the number of eggs produced between 2009 and 2015 was about twice higher (8.81 × 10^14^ to 1.45 × 10^15^ eggs; figure 7). From 2004 to 2006, the number of sardine eggs increased and was high (between 7.02 × 10^14^ and 1.47 × 10^15^ eggs for 2004–2006) but declined by one third until 2008 to then remain steady at low level (between 5.58 × 10^14^ and 5.28 × 10^14^ eggs for the 2008–2016 period; figure 7).

## 4. Discussion

Reproduction is costly, leading to a trade-off between reproductive investment, survival and growth [61]. The part of ingested energy remaining after allocation to metabolic process is allocated to both somatic growth and reproductive investments, which are hence in mutual competition. Recently, an increase of trophic overlap between small pelagic fish species was observed in the Gulf of Lions at the same time as a decrease in fish body condition [33,62], supporting the hypothesis that food resources could be currently more limiting than in the past [45,62]. In such context of food shortage, one might wonder how the trade-off between reproduction and growth or maintenance has been dealt with in both species, especially in light of their fast life-history pace as well as their opposite breeding strategies. To investigate reproductive allocation, we used a combination of three measurements: (i) the length of the breeding season, (ii) the age or size at which they first reproduce, and (iii) the weight of the gonad relative to the individual total weight.

First, the spawning period seems to have been slightly extended compared to previous studies from the 1960s [46,47]. In particular, anchovy starts reproducing a bit earlier, now starting at the end of April instead of in May. These changes could be the result of physiological adjustments to increasing sea temperatures and changing environment. Anchovy spawning was shown to be induced by temperatures higher than 17°C [34], so that advanced warming water [63] could promote earlier gonad maturation. As the reproductive performance is known to increase with age (constraint [64], restraint [5] and selection [65] hypotheses), especially in terms of breeding duration [61,66], spawning period was expected to be shorter for younger females than older ones [67,68]. Surprisingly, none of our studied species displayed a shorter spawning period despite a rejuvenation of the population, suggesting high reproductive investment for both species.

Furthermore, our results suggest a decrease in length at first maturity of both species. Sardine length at maturity was high during the 1970s and early 2000s, but has decreased strongly after 2009. An abrupt change in size at first maturity from 2007 to 2008 has also been observed in anchovy, which have matured at extremely small size since 2012. Surprisingly, the decline in *L*_50_ did not happen progressively [69], but very fast around 2008 in both species, confirming the high plasticity of their reproductive characteristics as already observed for other short-lived species, such as *Daphnia* [70], fish [71] or toad [72]. Under unfavourable environmental condition (reduced growth), organisms should adjust size at maturity to maximize fitness [73]. For instance, growth reduction often leads to earlier reproduction at a smaller size [74] in short-lived species. Similarly, under reduced adult survival, selection should favour genotypes capable of reproducing earlier, at a smaller size and with a higher reproductive effort [75]. Accordingly, sardine reproductive effort (as measured through the GSI) showed a strong increase during the last 7 years, anchovy GSI increasing as well though less strongly. Such increase in reproductive effort might be a response to the decreasing proportion of large females, which usually produce more eggs. Moreover, the gradual increase over the last years in sardine GSI (and also but more slowly so for anchovy) may reflect the progressive increase in the number of individuals able to invest highly in reproduction, supporting the idea that there was a selection favouring this phenotype.

Our results thus indicate that when confronted by low energy resources, small pelagic fish seem to increase reproductive allocation. This might be an important source of demographic changes as well as fishing pressure. As Van Beveren *et al*. [76] underlined the relatively low exploitation of both species during the study period in the study area, we do not think that fishing could be the main factor that has induced the observed changes in the studied indices. However, fishing can act as a covariable, and its impact might be difficult to differentiate from environmental ones. Fishing activities can largely affect the condition and reproductive potential of exploited fish in complex ways such as reducing the food availability or inducing physiological stress (reviewed by Lampert [15]), and therefore we cannot fully discard that fishing activities in the area targeting anchovy and sardine, despite being moderate, can play a role on the observed changes. Therefore, further investigations are needed to consider the fishing pressure on the condition and reproductive potential of small pelagic fish populations. By devoting more energy per individual to reproduction, small pelagic fish seem to favour their reproductive output over somatic growth under unfavourable conditions. These results support life-history theory, as short-lived species are expected to favour reproduction over survival [3], as previously shown in a large number of fish species (e.g. in vendace [71] or herring [77]). Contrary to longer-lived species able to safeguard their own survival by ceasing to breed at any time (e.g. amphibian [78] and reptile [79]), such a strategy could greatly affect other small pelagic life-history traits and explain the recent reduction of growth rates and sardine adult survival highlighted by Van Beveren *et al*. [33]. This might even be further amplified by the fact that the survival cost of reproduction is known to be higher in individuals maturing at smaller size and earlier age [80].

Despite similarities, our results also highlighted differences between the two species. Indeed, the increase in anchovy GSI values was much lower than sardine's one. This has to be put in relation with the steeper decline in growth and condition in sardines compared to anchovies, as well as the adult overmortality which only occurred in sardines [33]. Such results could come from their reproductive strategies, anchovy being an income breeder and sardine mainly a capital breeder [35,36]. If we assume that growth is mainly realized during spring and summer [81], when planktonic resources are more important, anchovy strategy allows them to take advantage of higher resource availability to invest in both somatic growth and reproduction. By contrast, sardine has to store energy and incur costs due to accumulating fat store and maintaining storage compound to then spend this capital during winter [82]. Following breeding at the end of winter, sardine reserves should thus be exhausted making them more vulnerable to lower food availability (whether due to a general decrease in prey quantity or quality). Together with really low energy stores, high reproductive investment at a period at which food is scarce could deeply lower the survival and prevent the majority of sardine from surviving past their first reproductive season, explaining the observed disappearance of large sardine since 2008 in the Gulf of Lions [31]. Nevertheless, some sardine populations were shown to feed during the spawning period while some anchovies rely on somatic reserves for part of their reproduction [42], smoothing off the income versus capital strategy opposition. Even if strict income or capital strategy might not happen all the time in the Gulf of Lions, our results still suggest potential differences in the effect of reproductive investment on other life-history traits depending on breeding strategies (income/capital), which would merit further investigation.

Results also suggest significant preovulatory maternal condition effects on the egg quantity and quality of sardine and anchovy. Reproductive features are similar to numerous species of birds [83], *Daphnia* [15], snakes [84] or turtles [14]. As the number of eggs increases linearly with fish size for both species and the RBF also increases with body length for sardine, reproductive capacity can therefore be assumed to be higher in large individuals. This emphasizes the hypothesis that the reproductive potential of a species is highly dependent on large individuals (i.e. dependent on the age structure of the population, e.g. [85]) as previously reported for many taxa (e.g. [86]). However, contrasting with several other species, e.g. birds [25] or reptiles [22], anchovy and sardine females in better condition did not produce more eggs relative to their weight than females in a poorer condition. On the contrary, egg quality did depend on female lipid content, so that maternal condition may be relevant for the survival of egg and larvae. Indeed, Riveiro *et al*. [87] demonstrated the link between larval survival in hatching condition and the egg quality underlining its importance in larvae survival rate and in short-term fish recruitment. Consequently, we may reasonably think that later years' egg quality of sardine and anchovy was affected by the decrease of adult body condition [33]. While a positive size effect had been previously detected on egg quality in cod [88] but also in turtles [14] or birds [89], none of our species displayed such relationship. Moreover, the occurrence and intensity of atresia were not related to fish condition or size in either of the two species, despite maternal condition being know to rule oocytes resorption mechanism depending on fat quantity in several species, e.g. in insects [90] or fish [91]. This could be due to the really low level of atresia observed in these indeterminate fecundity species, for which atresia occurs almost only in the regression phase.

In the light of the current small pelagic fish situation in the Gulf of Lions, characterized by small sardine and anchovy in poor condition [33,54], our results indicate that the individual reproductive potential could be strongly affected both in terms of quantity and quality. However, earlier maturation could potentially lead to a higher number of breeders and compensate at the population level for the decrease in individual reproductive capacity, as described in other short-lived species such as *Daphnia* [92] or insects [93]. Indeed, the back-calculated yearly population egg number production values indicated that anchovy egg production has been slightly higher since 2009 and thus not affected by low resource levels and smaller fish dominance. On the contrary, the change in *L*_50_ was not sufficient to counteract the more pronounced disappearance of large and old individuals in sardines. Indeed, the model highlighted an estimated fourfold reduction of the sardine egg number in the Gulf of Lions between years when large sardines dominated (i.e. 2005–2006) and when small sardines dominated (i.e. since 2008). As sardine and anchovy condition has decreased since 2007 and influences egg quality, we suggest that along with egg number reduction, egg quality also decreased. This enhances the idea of a stronger degradation of sardine reproductive capacities paralleling the decline in the lipid reserves of the stock compared to anchovy. Nevertheless, no data were currently available to obtain an accurate estimation of recruitment for both species, leaving as a challenge for future studies to test whether egg number and quality might explain a significant part of recruitment variability of small pelagic fish.

Long-lived species are able to prioritize their energy allocation between life-history traits and usually favour their own condition over their propagules' condition [3]. For example, fish [42] as well as birds [94] or reptiles [95] are able to skip or delay breeding under poor environmental condition to attempt to maximize fitness by allocating resources optimally among growth, maintenance and future reproduction. In contrast, short-lived anchovy and sardine clearly guided the trade-offs between reproduction and survival towards a maintenance (if not an increase) of their reproductive investment even during a poor condition period in the Gulf of Lions. According to the costs associated with reproduction, females favouring reproduction led to a reduced growth and a reduction in survival and might explain the current lack of large old small pelagic fish in the Gulf of Lions. Even if reproduction was prioritized, egg quantity and quality decreased between 2006 and 2015 for sardine. While the effect of decreasing sardine egg production on its recruitment could not be investigated in this study, these findings brought to light strong evidence on the need to consider fish reproductive and condition characteristics together. Both are essential to understand forage fish fluctuations and evaluate the long-term sustainability of the forage fish stocks.

## Data accessibility

The dataset used in this study is available at Dryad repository under accession number http://dx.doi.org/10.5061/dryad.6jk98 [96] and can be used and cited from this link.
